# Supplementary figures and images for: Cyperus rotundus Extract and Its Active Metabolite α-Cyperone Alleviates Paclitaxel-Induced Neuropathic Pain via the Modulation of the Norepinephrine Pathway
Source: Metabolites. 2024 Dec 20;14(12):719. doi: 10.3390/metabo14120719 (PMC11679560; doi:10.3390/metabo14120719)

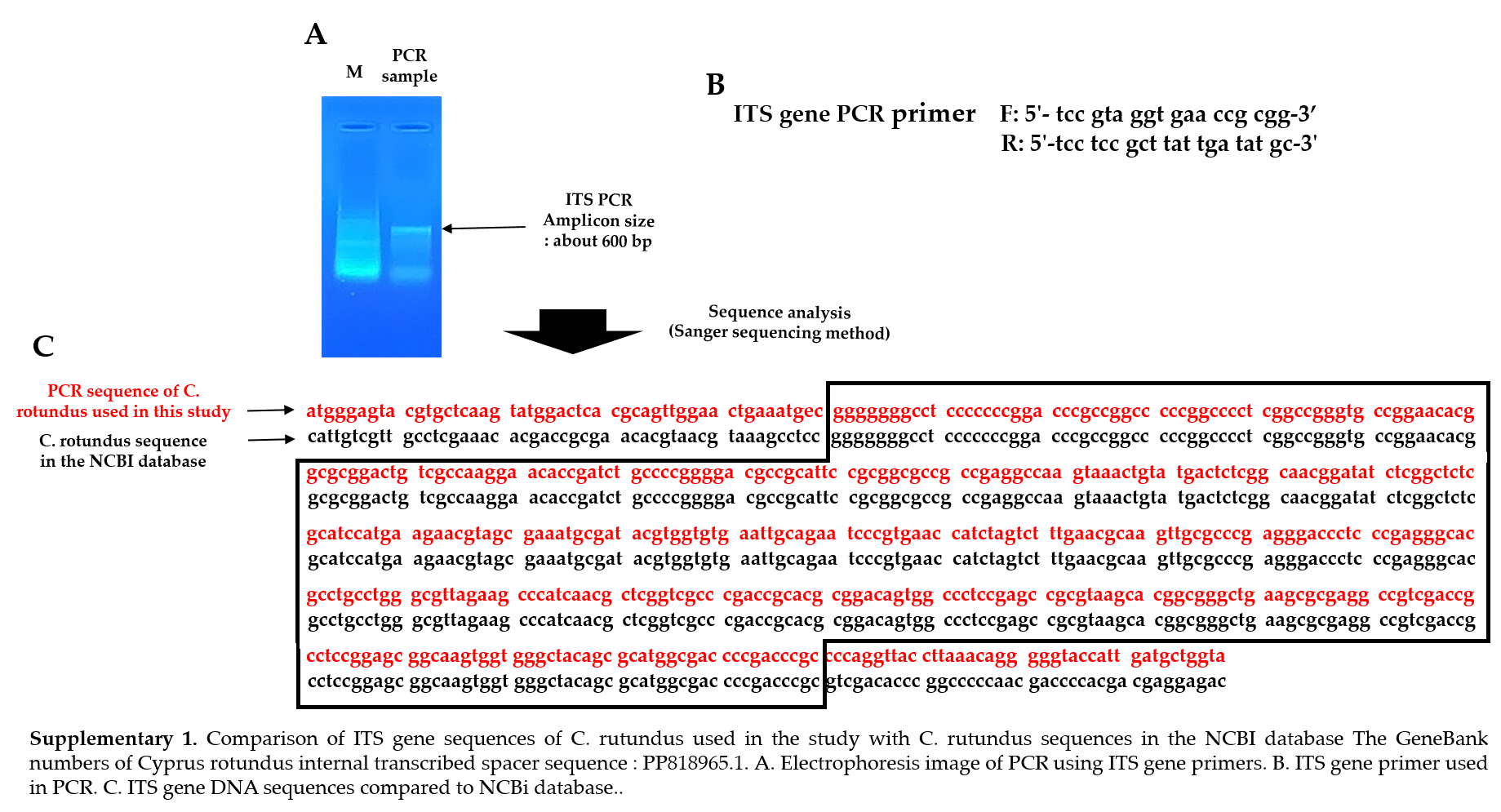

Supplement: Supplementary file 1 [file metabolites-14-00719-s001.zip › metabolites-3316893-supplementary-File S1.png]

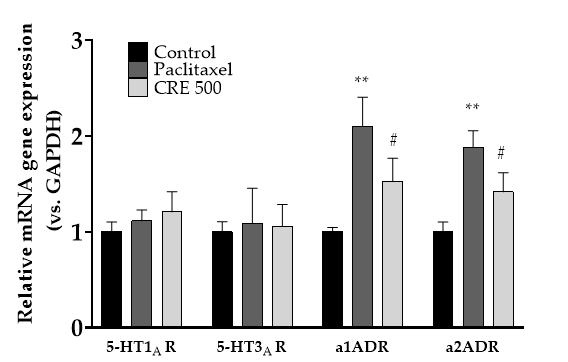

Supplement: Supplementary file 1 [file metabolites-14-00719-s001.zip › metabolites-3316893-supplementary-File S2.png]
